# HSPB5 suppresses renal inflammation and protects lupus-prone NZB/W F1 mice from severe renal damage

**DOI:** 10.1186/s13075-022-02958-9

**Published:** 2022-12-12

**Authors:** Justin Knapp, Marsela Braunstein, Spencer Iner Thomas Berg, Cody Shirriff

**Affiliations:** Serenity Bioworks, 151 Charles St. W, Suite 199, Kitchener, ON Canada

**Keywords:** Lupus nephritis, Systemic lupus erythematosus, Autoimmunity, Heat shock proteins, HSPB5, NZB/W F1 mice, Macrophages, Inflammation, Treg, Breg

## Abstract

**Background:**

Lupus nephritis (LN) is an inflammatory disease of the kidneys affecting patients with systemic lupus erythematosus. Current immunosuppressive and cytotoxic therapies are associated with serious side effects and fail to protect 20–40% of LN patients from end-stage renal disease. In this study, we investigated whether a small heat shock protein, HSPB5, can reduce kidney inflammation and the clinical manifestations of the disease in NZB/W F1 mice. Furthermore, we investigated whether HSPB5 can enhance the effects of methylprednisolone, a standard-of-care drug in LN, in an endotoxemia mouse model.

**Methods:**

NZB/W F1 mice were treated with HSPB5, methylprednisolone, or vehicle from 23 to 38 weeks of age. Disease progression was evaluated by weekly proteinuria scores. At the end of the study, the blood, urine, spleens, and kidneys were collected for the assessment of proteinuria, blood urea nitrogen, kidney histology, serum IL-6 and anti-dsDNA levels, immune cell populations, and their phenotypes, as well as the transcript levels of proinflammatory chemokine/cytokines in the kidneys. HSPB5 was also evaluated in combination with methylprednisolone in a lipopolysaccharide-induced endotoxemia mouse model; serum IL-6 levels were measured at 24 h post-endotoxemia induction.

**Results:**

HSPB5 significantly reduced terminal proteinuria and BUN and substantially improved kidney pathology. Similar trends, although to a lower extent, were observed with methylprednisolone treatment. Serum IL-6 levels and kidney expression of BAFF, IL-6, IFNγ, MCP-1 (CCL2), and KIM-1 were reduced, whereas nephrin expression was significantly preserved compared to vehicle-treated mice. Lastly, splenic Tregs and Bregs were significantly induced with HSPB5 treatment. HSPB5 in combination with methylprednisolone also significantly reduced serum IL-6 levels in endotoxemia mice.

**Conclusions:**

HSPB5 treatment reduces kidney inflammation and injury, providing therapeutic benefits in NZB/W F1 mice. Given that HSPB5 enhances the anti-inflammatory effects of methylprednisolone, there is a strong interest to develop HSBP5 as a therapeutic for the treatment of LN.

**Supplementary information:**

The online version contains supplementary material available at 10.1186/s13075-022-02958-9.

## Background

Lupus nephritis (LN) affects nearly 50% of adults and 80% of children diagnosed with systemic lupus erythematosus (SLE), a chronic autoimmune and inflammatory disease [1]. The current standard of care includes high-dose intravenous glucocorticoid followed by oral prednisone tapers and cyclophosphamide or mycophenolate mofetil (MMF) [2]. Although improvements in LN outcomes have been observed over the past several decades, the risk of kidney failure within 15 years of LN diagnosis is 22% for all LN classes and 44% for class IV LN [3]. These outcomes could improve further with recent approvals of targeted therapies. Ideally, treatment combinations should focus on suppressing intrarenal inflammation for immediate relief of LN, and autoimmunity to delay subsequent LN flares [4].

Heat shock proteins (HSP) perform pleiotropic functions. Intracellularly, HSPs function as molecular chaperones to prevent protein aggregation during cellular stress [5–7]. HSPs can also be secreted into the extracellular environment as a free protein or within exosomes [8–10], where they act as potent regulators of inflammation. HSPs mediate signaling via innate immune receptors such as CD14; Toll-like receptors (TLRs) 1, 2, or 4; or scavenger receptors on various cells including macrophages, dendritic cells, and kidney tubular cells [11–15].

Of particular interest is the small HSP ɑB-crystallin (HSPB5), which was originally described as a major component of the eye protecting against lens protein aggregation [6] but has since been discovered as being abundantly expressed in other tissues including the kidneys [16, 17]. Similar to many other HSPs, HSPB5 has been demonstrated to be released into the extracellular space during cellular stress to reduce inflammation and tissue damage [11, 18]. Similarly, exogenously administered HSPB5 has been shown to limit the extent of damage in rodent models of stroke [19], spinal cord injury [20], and experimental autoimmune encephalitis [21, 22]. The protective effects of exogenous HSPB5 are likely primarily mediated through their well-described effects on macrophages. It has been demonstrated in pre-active lesions from multiple sclerosis patients that stressed oligodendrocytes secrete abundant HSPB5, which binds nearby microglia via CD14 and TLR1/2 to induce dramatic changes in the gene expression, including the induction of many anti-inflammatory cytokines [11]. Furthermore, macrophages from mice deficient in HSPB5 are hyperactive and secrete substantially increased amounts of inflammatory cytokines (IL-1β, IL-12p40, TNF, and IL-6) upon stimulation with lipopolysaccharide (LPS) [21].

Macrophages are key regulators of inflammation, immunity, and tissue homeostasis with a wide variety of activities and presence in a broad range of tissues. Upon inflammatory injury, resident and infiltrating macrophages may be differentiated and activated and often take on pathogenic roles in autoimmune settings. Macrophages are dysfunctional in SLE and LN in a number of ways, including deficient clearance of apoptotic cells, abnormal polarization, secretion of inflammatory mediators, and tissue destruction through the expression of proteolytic enzymes [23]. Furthermore, these dysfunctions may drive aberrant lymphocyte activity in SLE [24]. Therapies that restore normal macrophage function may therefore provide substantial benefit to SLE patients.

Because of the potent anti-inflammatory effects of HSPB5 that are mediated through macrophages and the involvement of these cells in SLE/LN pathology, we hypothesized that HSPB5 could provide a therapeutic effect in SLE by reducing the aberrant activation and inflammatory signaling of macrophages. We have previously demonstrated the protective effects of HSPB5 treatment in the MRL/lpr mouse model of SLE and in particular showed its ability to reduce the extent of LN by limiting renal damage [25]. In this study, we used NZB/W F1 mice, a classic model of SLE with aberrant innate signaling and macrophage activity, to compare the efficacy of HSPB5 to a standard of care SLE medication, methylprednisolone, and to investigate the immune cell and inflammatory marker changes in the kidneys during HSPB5 treatment.

## Methods

### Test articles

Recombinant HSPB5 was manufactured by Delta Crystallon, and negligible endotoxin levels (< 0.05 EU/mg) were confirmed as previously described [25]. Methylprednisolone was purchased from Sigma-Aldrich (M3781).

### Animals

Mice were purchased from the Jackson Laboratories and housed under pathogen-free conditions at the animal research facility of the University of Waterloo (Waterloo, ON, Canada). All procedures involving mice were approved by the Office of Research Ethics and animal care committee at the University of Waterloo (AUPs 41798 and 43991). Mice were acclimated for at least 4 weeks prior to the study start.

### SLE study design

At 23 weeks of age, female NZB/W F1 mice (strain #100008) were evenly distributed by body weight and proteinuria score into three groups with 14–15 mice per group. Mice were treated three times weekly for 15 weeks by intraperitoneal injection (IP) with HSPB5 (0.3 mg/kg), methylprednisolone (3 mg/kg), or PBS as a vehicle control, at a volume of 50 µL per 10 g body weight. Disease progression was monitored by body weight and condition, behavior, and weekly measurements of urinary protein content (proteinuria). Treatments and scoring were performed by animal health technicians (AHTs) blinded to the experimental groups. Mice were terminated upon reaching 38 weeks of age or upon the recommendation of AHTs and the consulting veterinarian after achieving humane endpoints (proteinuria ≥ 3, poor body condition, body weight loss > 15%), and tissues (blood, spleens, and kidneys) were collected for further analysis.

### Endotoxemia study design

At 11 weeks of age, male BALB/c mice (strain #00065) were evenly distributed by body weight into 4 groups with 11 mice per group. Endotoxemia was induced by IP injection of 6 mg/kg LPS (O111:B4, Sigma-Aldrich, L4391). Mice were treated 30 min before and 6 h after the LPS challenge, by IP injection with HSPB5 (0.3 mg/kg), methylprednisolone (1.5 mg/kg), HSPB5 (0.3 mg/kg) + methylprednisolone (1.5 mg/kg), or PBS as a vehicle control at a volume of 80 µL per 10 g body weight. Mice were euthanized 24 h post-LPS injection, and the serum was collected for further analysis.

### Assessment of renal function

Proteinuria was measured using a commercial dipstick test (Siemens; Uristix) and scored semi-quantitatively as follows: 0 (no/trace protein), 1 (30 mg/dL), 2 (100 mg/dL), 3 (300 mg/dL), and 4 (≥ 2000 mg/dL). Half scores were assigned when the color was intermediate between two whole values. Scoring was performed by AHTs blinded to the study. Blood urea nitrogen (BUN) was measured from terminal serum samples using a commercial kit (Thermo Fisher) as per the manufacturer’s instructions.

### Histology

The whole kidneys and spleen portions were fixed in 10% formalin and embedded in paraffin. Two sections (top and middle) were cut for staining with hematoxylin and eosin (H&E). The kidneys were additionally cut for periodic acid–Schiff (PAS) staining. Slides were scanned using TissueScope LE120 digital slide scanner (Huron Digital) at 20 × and evaluated by an experienced veterinary pathologist blinded to the study. The severity of glomerular deposits, mesangial expansion, endocapillary proliferation, extracapillary proliferation, tubular atrophy, and interstitial infiltrates was graded semi-quantitatively: 0 (no change), 1 (mild), 2 (moderate), and 3 (severe). The total glomerular lesions score represents the sum of individual scores for glomerular deposits, mesangial expansion, endocapillary proliferation, and extracapillary proliferation. The total kidney lesion score represents the sum of all individual scores [26].

#### ELISA

IL-6 was measured from terminal serum samples using a commercial ELISA kit (BD Biosciences; Mouse IL-6 ELISA Set) as per the manufacturer’s instructions. Anti-dsDNA antibody titers were measured from terminal serum samples using a commercial ELISA kit (Signosis; Mouse Anti-dsDNA ELISA Kit) as per the manufacturer’s instructions.

### qRT-PCR

Kidney portions were stored in RNAlater™ (Thermo Fisher) until analysis. RNA was isolated using a commercial kit (Promega; Reliaprep™ RNA Miniprep) as per the manufacturer’s instructions and reverse transcribed with GoScript™ Reverse Transcription System kit using random primers (Promega). Reactions were run on 10-ng RNA equivalents in triplicate with Fast Advanced MasterMix (Thermo Fisher) and TaqMan™ probe and primer sets (Thermo Fisher): CCL2 (Mm00441242_m1), IFNγ (Mm01168134_m1), IL-6 (Mm00446190_m1), IL-10 (Mm01288386_m1), TNF (Mm00443258_m1), Nphs1 (Mm01176615_g1), Havcr1 (Mm00506686_m1), BAFF (Mm00446347_m1), and TATA-Binding Protein (TBP; Mm00446973_m1) as an endogenous control. Probes against genes of interest were labeled with FAM, whereas the TBP probe was labeled with VIC. Data were analyzed using the 2^−ΔΔCT^ method as previously described [27]. Statistical analyses were performed on log-transformed data.

### Cell isolation and flow cytometry

A portion of the spleen and kidney was processed to single cells for flow cytometric analysis. Spleen fragments were dissociated using a plunger from a sterile 1-mL syringe (BD) and a 40-µm strainer (Bio Basic). Kidney pieces were digested in complete DMEM media (2 mM l-glutamine, 50 µM β-mercaptoethanol (Thermo Fisher), 25 mM HEPES (Thermo Fisher), 1% penicillin–streptomycin (Sigma Aldrich), 1 mM sodium pyruvate (Thermo Fisher), and 1 × MEM non-essential amino acids (Thermo Fisher)) with 1 mg/mL collagenase IV (Thermo Fisher) for 30 min at 37 °C. Kidney pieces were further mechanically disrupted by sequential pipetting using 25 mL, 10 mL, and 5 mL serological pipettes, then passed through the 40-µm strainer as described for the spleens. Both spleen and kidney single-cell suspensions were incubated with RBC lysis buffer (Millipore Sigma) for 3 min, then prepared for flow cytometric analysis. Briefly, cells were incubated with Fc block (2.4G2, STEMCELL Technologies) for 10 min, followed by a 20-min staining with fluorochrome-conjugated antibodies: CD45-PE (30-F11, BD Biosciences), CD11b-FITC (M1/70, BD Biosciences), F4/80-PerCP-Cy5.5 (BM8, Thermo Fisher), Ly6G-PE-Cy7 (1A8, BD Biosciences), Ly6C-APC (B-ly6, BD Biosciences), CD11c-APC-R700 (N418, BD Bioscience), CD86-PE-Cy7 (GL1, BD Biosciences), CD206-AlexaFluor700 (MR6F3, BD Biosciences), Siglec-1-APCeFluor780 (SER-4, Thermo Fisher), CD3-FITC (145-2C11, BD Biosciences), CD4-PE (GK1.5, R&D Systems), CD25-PerCP-Cy5.5 (PC61, BD Biosciences), CD19-PE-Cy7 (1D3, BD Biosciences), and CD5-APC-R700 (53–7.3, BD Biosciences). For intracellular staining, cells were fixed and permeabilized using eBioscience Intracellular Fixation & Permeabilization Buffer Set (Thermo Fisher) followed by a 30-min staining with anti-Arg1-APC (A1exF5, Fisher Scientific) and anti-FoxP3-APC antibodies (1054C, R&D Systems). Events were acquired using Attune NxT cytometer (Thermo Fisher) and analyzed using FlowJo v.10.8 (BD Biosciences).

### Statistical analysis

Data are presented as means ± standard error of the mean (SEM) unless otherwise indicated in the figure legends. All data were assessed for normality by the Shapiro–Wilk test. Data following a normal distribution were analyzed by ordinary one-way ANOVA and corrected for multiple comparisons using Dunnett’s test (> 2 groups) or by Student’s *t*-test (2 groups). Data that did not follow a normal distribution were analyzed by the non-parametric Kruskal–Wallis test and corrected for multiple testing using Dunn’s test (> 2 groups) or the Mann–Whitney test (2 groups). The Pearson correlation coefficient was used to determine the relationship between kidney function (proteinuria, BUN) and pathology scores (total glomerular lesions, total kidney lesions). Data were analyzed in GraphPad Prism 9.0.0. The results were considered statistically significant when *p*-values were less than 0.05.

## Results

### HSPB5 improves renal function and survival in NZB/W F1 mice

**Fig. 1:**
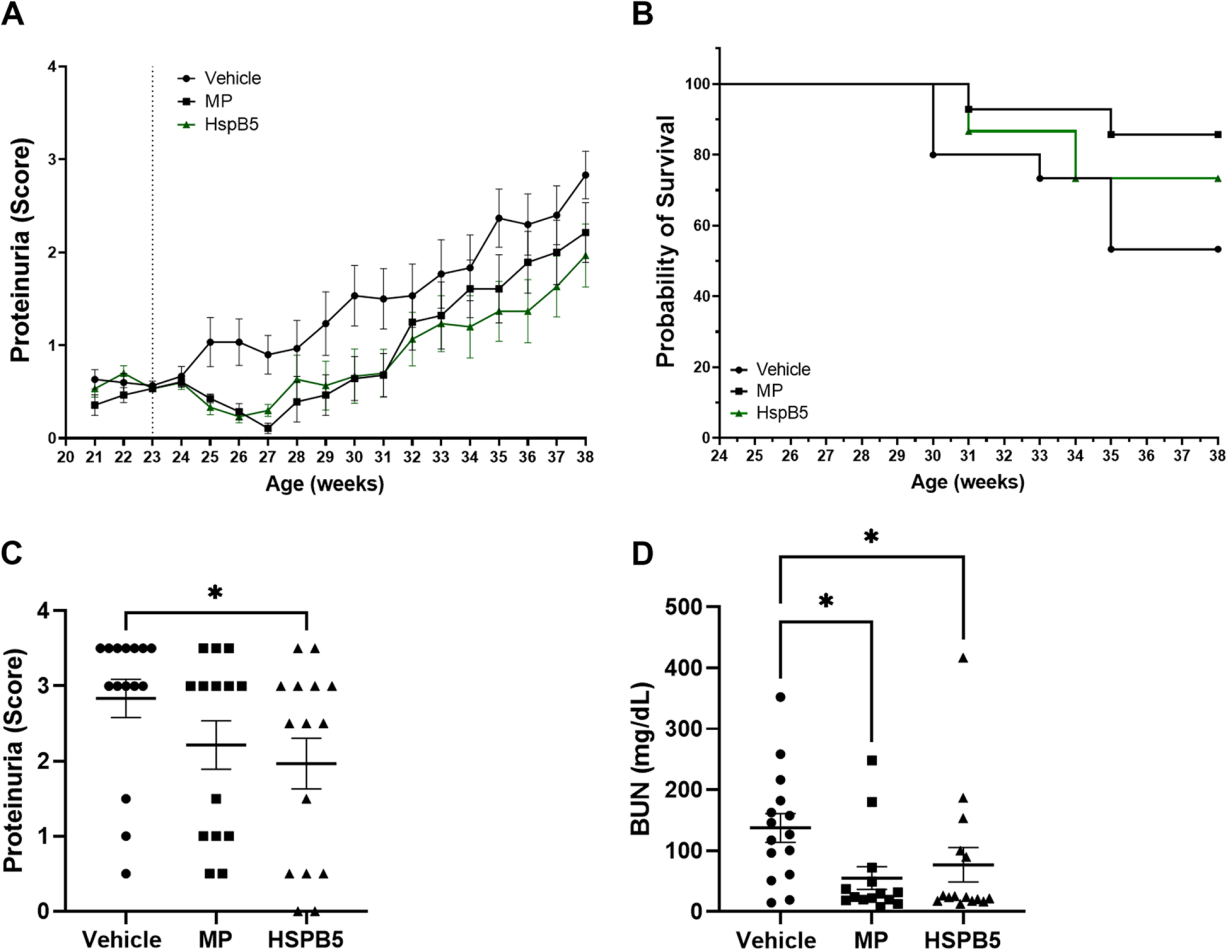
HSPB5 improves renal function and survival in NZB/W F1 mice. Female NZB/W F1 mice of 23 weeks of age were treated intraperitoneally with 0.3 mg/kg HSPB5, 3 mg/kg methylprednisolone (MP), or vehicle until week 38 (*n* = 14–15 per group). **A** Proteinuria over time. **B** Kaplan–Meier curve showing survival per treatment group. **C** Terminal proteinuria levels. **D** Blood urea nitrogen (BUN) levels measured from terminal serum samples. *p < 0.05. **p < 0.01

Given that our previous study with HSPB5 demonstrated attenuated LN in the MRL/lpr lupus mouse model [25], we were interested in comparing the efficacy of HSPB5 to a glucocorticoid. In the present study, we chose to examine the protective effects of HSPB5 in NZB/W F1 mice, a genetically aligned mouse model of human LN. NZB/W F1 mice were injected IP with 0.3 mg/kg HSPB5 (an average dose of 13.5 µg HSPB5 per mouse), 3 mg/kg methylprednisolone, or vehicle, three times per week, starting at 23 weeks of age. At this age, NZB/W F1 mice have well-established autoimmunity as evident by high titers of anti-dsDNA antibodies, but still have little renal damage as evident by low to negligible urinary protein, making them comparable to LN/SLE patients with low disease activity [28]. Vehicle-treated mice developed severe disease marked by progressive proteinuria, body weight loss, and poor survival, as expected in this model. In contrast, HSPB5-treated mice maintained negligible proteinuria (average score < 1) from weeks 23 to 31 and the lowest proteinuria levels among all groups until week 38 (Fig. 1A); furthermore, they gained body weight and had marginally improved survival when compared to the vehicle group but was not statistically significant (Additional file 1: Figure S1A; Fig. 1B). Importantly, HSPB5-treated mice showed significantly lower terminal proteinuria scores and BUN levels (Fig. 1C, D). Methylprednisolone treatment also showed significantly lowered terminal BUN levels but did not show significantly lower terminal proteinuria scores (Fig. 1C, D).

### HSPB5 treatment reduces terminal serum IL-6 but not anti-dsDNA titers

**Fig. 2:**
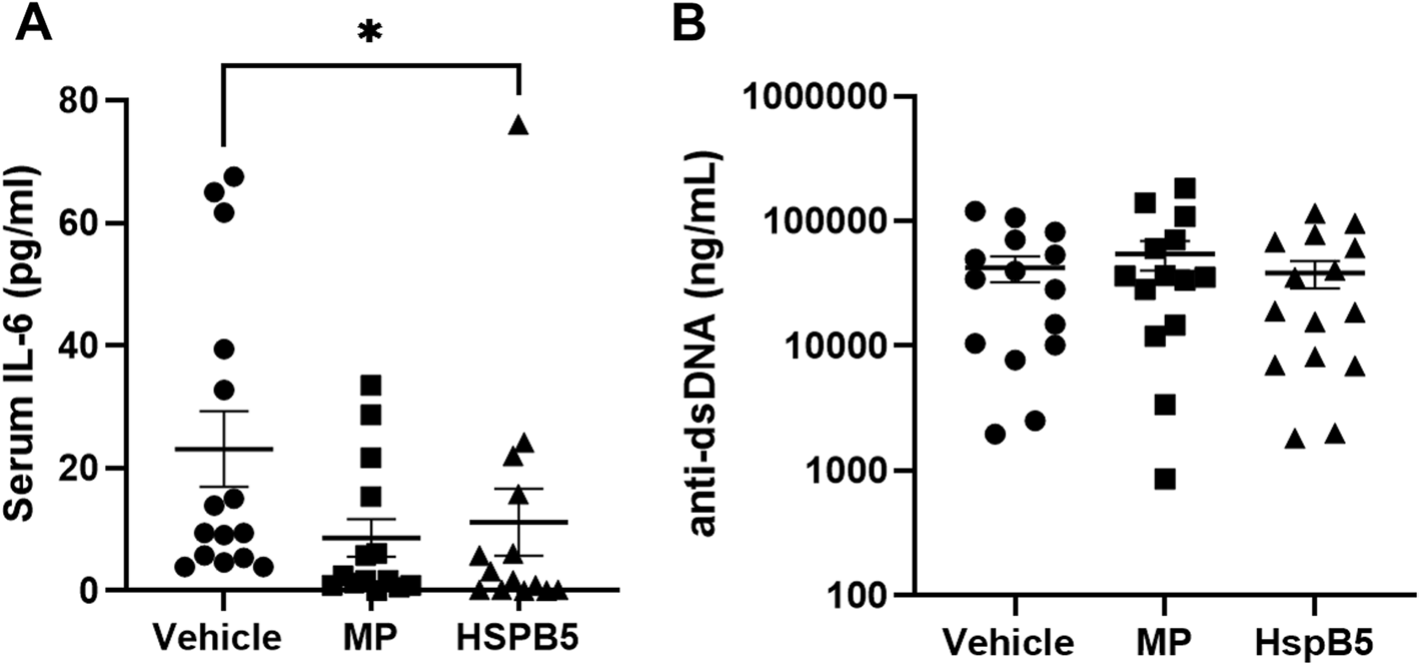
HSPB5 reduces terminal serum IL-6 but not anti-dsDNA titers. Female NZB/W F1 mice of 23 weeks of age were treated intraperitoneally with 0.3 mg/kg HSPB5, 3 mg/kg methylprednisolone (MP), or vehicle until week 38 (*n* = 14–15 per group). At 38 weeks, mice were sacrificed for blood collection. **A** Serum IL-6 levels as determined by ELISA. **B** Serum anti-dsDNA antibody levels determined by ELISA. *p < 0.05. **p < 0.01

Progression of proteinuria in NZB/W F1 mice and disease activity in SLE patients are associated with elevated IL-6 levels [29]. Thus, we measured the concentration of IL-6 in terminal serum samples by ELISA. HSPB5 treatment significantly reduced serum IL-6 levels compared to vehicle-treated mice (Fig. 2A). Serum IL-6 levels were also reduced with methylprednisolone but did not reach statistical significance (Fig. 2A). Since IL-6 plays an important role in the generation of autoantibodies, we also examined the levels of anti-dsDNA antibodies in the serum samples. While there was no effect on terminal anti-dsDNA antibody titers (Fig. 2B), HSPB5 treatment modestly delayed anti-dsDNA antibody titers early in the study, at week 27 (Additional file 1: Figure S2).

### Treatment with HSPB5 reduces immune activation and promotes tolerance induction in the spleen

**Fig. 3:**
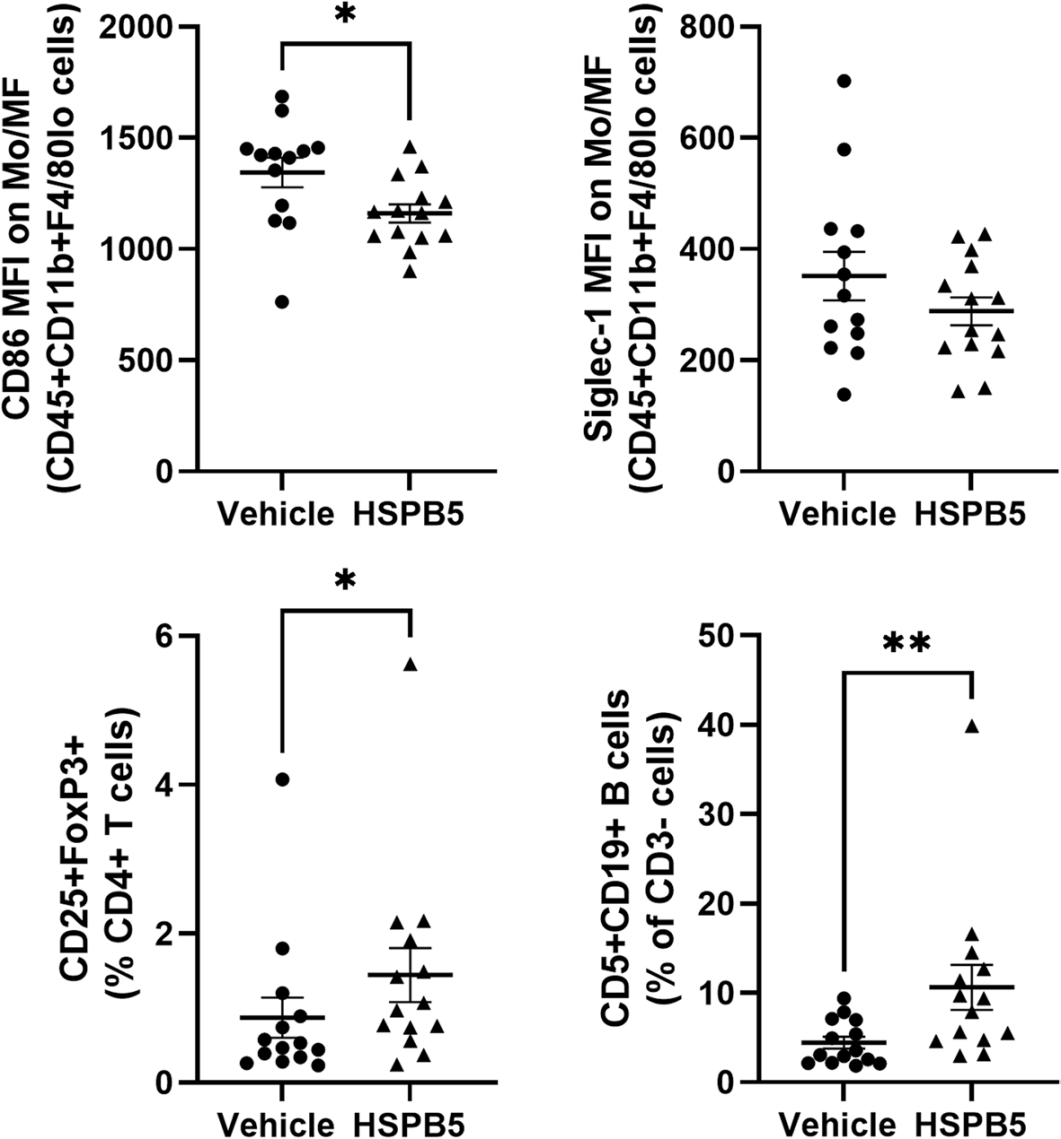
Treatment with HSPB5 reduces immune activation and promotes tolerance. Female NZB/W F1 mice of 23 weeks of age were treated intraperitoneally with 0.3 mg/kg HSPB5, 3 mg/kg methylprednisolone (MP), or vehicle until week 38 (*n* = 14–15 per group). At 38 weeks, mice were sacrificed for tissue collection. Flow cytometric analysis of splenocytes: CD86 and Siglec-1 mean fluorescence intensity (MFI) on CD45 + CD11b + F4/80lo cells; Tregs (CD25 + FoxP3 + cells shown as a percent of CD3 + CD4 + T cell population) and Bregs (CD5 + CD19 + cells shown as a percent of CD3 − population). *p < 0.05. **p < 0.01

The observed improvement in renal function prompted us to examine whether HSPB5 played an immunoregulatory role in the spleen, where pathogenic lymphocytes are activated and expanded by APCs presenting autoantigens. To assess the effect of HSPB5 treatment on APC and lymphocyte activation in the spleen, we performed flow cytometry. HSPB5 had no effect on the distribution of T cells (CD3 +), B cells (CD19 +), myeloid cells (CD11b +), neutrophils (CD11b + Ly6G +), or mDCs (CD11b + CD11c +) (data not shown). Myeloid cells were further evaluated for the expression of activation markers, CD86, and Siglec-1. Compared to the vehicle group, mice treated with HSPB5 had decreased expression of CD86 and Siglec-1 on splenic monocytes/macrophages (CD11b + F4/80lo) (Fig. 3). T cell and B lymphocytes were further profiled for subpopulations; there was no difference in the frequencies of CD4 + and CD8 + cells (data not shown). However, regulatory T cells (Tregs; CD25 + FoxP3 +) and regulatory B cells (Bregs; CD5 + CD19 +) were significantly increased in the spleens of HSPB5-treated mice (Fig. 3).

### HSPB5 reduces the infiltration of immune cells into the kidney and prevents severe kidney damage

**Fig. 4:**
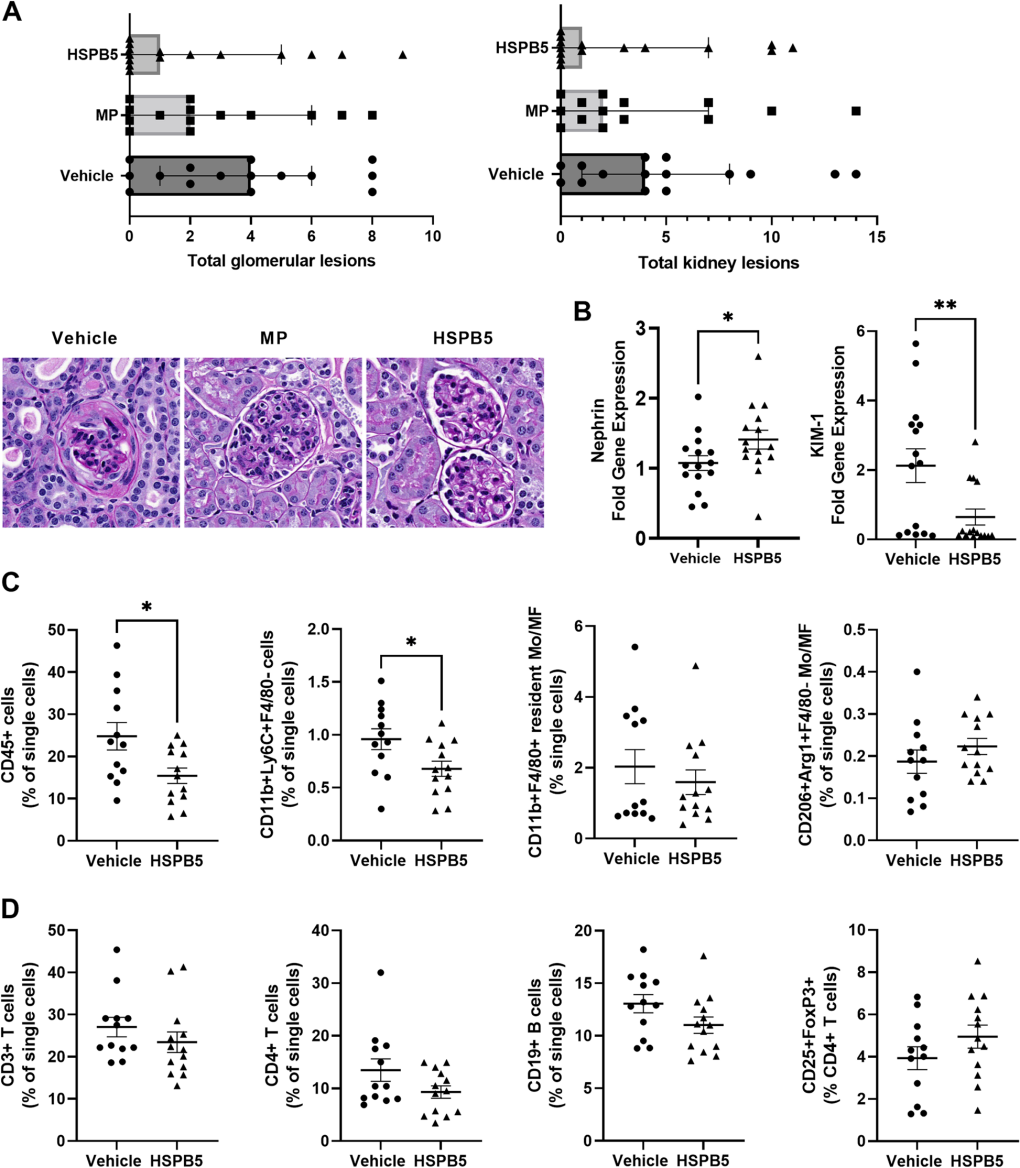
HSPB5 reduces the infiltration of immune cells into the kidney and improves kidney pathology. Female NZB/W F1 mice of 23 weeks of age were treated intraperitoneally with 0.3 mg/kg HSPB5, 3 mg/kg methylprednisolone (MP), or vehicle until week 38 (*n* = 14–15 per group). At 38 weeks, mice were sacrificed for tissue collection. **A** The kidneys were formalin-fixed, paraffin-embedded (FFPE) and stained with periodic acid–Schiff (PAS) prior to evaluation by a pathologist blinded to the study. Top: total glomerular lesions and kidney lesions shown as median + 95% confidence intervals. Bottom: glomerular morphology at 20 × from representative mice from each experimental group. **B** qRT-PCR analysis of kidney cells. The graphs show the mean fold changes in gene expression ± SEM. Statistical analysis was performed on log-transformed data. **C** The kidneys were processed as described in detail in the “Methods” section. Single-cell suspensions were stained and analyzed by flow cytometry for total leukocytes (CD45 +), infiltrating inflammatory macrophages (CD11b + Ly6C + F4/80 −), resident macrophages (MF; CD11b + F4/80 +), and M2-like monocytes/macrophages (Mo/MF; CD206 + Arg-1 + F4/80 −). **D** Flow cytometric analysis of lymphocyte populations defined as total T cells (CD3 +), CD4 + T cells, total B cells (CD19 +), and Tregs (CD25 + FoxP3 +, shown as a percent of CD4 + T cells). *p < 0.05. **p < 0.01

Upon histological examination of the kidneys, mice in the control group showed substantial glomerular lesions, particularly glomerular deposits and mesangial proliferation (Fig. 4A, Additional file 1: Figure S3). Consistent with this disease model, we also observed endocapillary proliferation, tubular atrophy, and interstitial infiltrates (Additional file 1: Fig. S3). HSPB5 treatment substantially improved kidney pathology as evident by reduced severity of total glomerular lesions and reduced overall kidney lesion scores (Fig. 4A). Additionally, HSPB5 treatment trended towards reduced tubular atrophy (an indicator of chronic renal inflammation), glomerular deposits, mesangial expansion, and endocapillary proliferation (Additional file 1: Figure S3). Importantly, reduced proteinuria correlated with reduced total glomerular lesions (*r* = 0.7, *p* = 0.008) and reduced total kidney lesions (*r* = 0.7, *p* = 0.007) in the HSPB5 group. Similarly, the decrease in BUN correlated with improved total glomerular lesion scores (*r* = 0.9, *p* < 0.0001) and total kidney lesion scores (*r* = 0.8, *p* = 0.001) in HSPB5-treated mice. Treatment with methylprednisolone also alleviated kidney pathology, but the extent of the improvement was greater in HSPB5-treated mice (Fig. 4A, Additional file 1: Figure S3). In agreement with the reduced severity of kidney damage, mice treated with HSPB5 significantly retained the expression of nephrin, a podocyte junction protein whose loss is involved in proteinuria development [30] and significantly decreased the expression of the tubular injury marker, KIM-1, suggesting preserved tubular integrity (Fig. 4B). Of note, there was a slight, but non-significant decrease in the total weight of the kidneys from these mice (Additional file 1: Figure S1B).

Next, we performed flow cytometric analysis on total kidney cells to compare the extent of leukocyte infiltration and activation between vehicle and HSPB5-treated mice. The frequency of leukocytes within the kidneys of HSPB5-treated mice was significantly decreased compared to the vehicle group (Fig. 4C). Upon examination of the myeloid cells, we found that HSPB5 treatment significantly reduced the infiltration of inflammatory monocytes/macrophages (CD11b + Ly6C + F4/80 −) (Fig. 4C). Resident macrophages (CD11b + F4/80 +) were also decreased, whereas M2-like Mo/MF (CD206 + Arg-1 + F4/80 −) were slightly elevated, although non-significantly (Fig. 4C). The frequency of mDCs (CD11b + CD11c +), neutrophils (CD11b + Ly6G +), and activated macrophages (CD86 + or Siglec-1 +) were unchanged (Additional file 1: Figure S4). Within the lymphoid cell populations, we observed slight reductions in total T cells (CD3 +), Th cells (CD4 +), and total B cells (CD19 +) (Fig. 4D), consistent with the lower infiltration of the kidneys by the immune cells (Fig. 4C). Tregs were slightly elevated in the HSPB5 group (ns; Fig. 4D); no changes were observed in CD8 + T cells or Bregs (Additional file 1: Figure S4).

### Treatment with HSPB5 suppresses renal inflammation

**Fig. 5:**
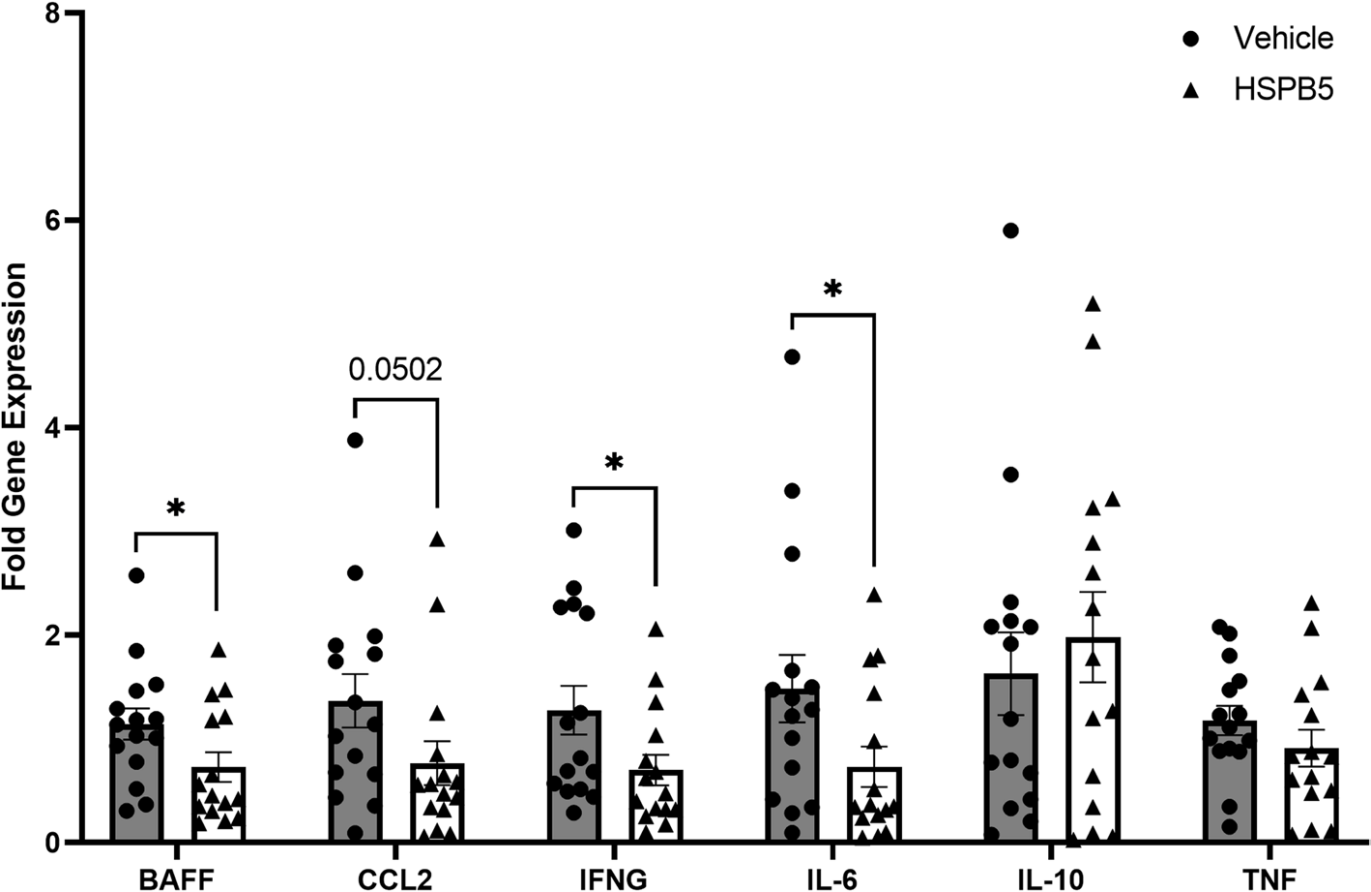
Treatment with HSPB5 suppresses renal inflammation. qRT-PCR of total kidney cells. Individual and mean fold changes in the gene expression ± SEM. Statistical analysis was performed on log-transformed data. *p < 0.05. **p < 0.01. *p*-value for CCL2 is shown to appreciate the difference between vehicle and HSPB5 treatments

To further characterize the functional implications of the observed changes in renal architecture and leukocyte populations, we performed qRT-PCR on total kidney cells. We examined the expression of IL-10 and key inflammatory markers of LN progression: IFNγ, IL-6, TNF, CCL2 (MCP-1), and BAFF. No major changes were observed in the expression of TNF and IL-10 between the vehicle and HSPB5 groups (Fig. 5). CCL2 was substantially lower, but it did not reach statistical significance (*p* = 0.0502). Importantly, IFNγ, IL-6, and BAFF were significantly reduced in HSPB5-treated mice (Fig. 5).

### HSPB5 enhances an anti-inflammatory activity of methylprednisolone in an endotoxemia mouse model

**Fig. 6:**
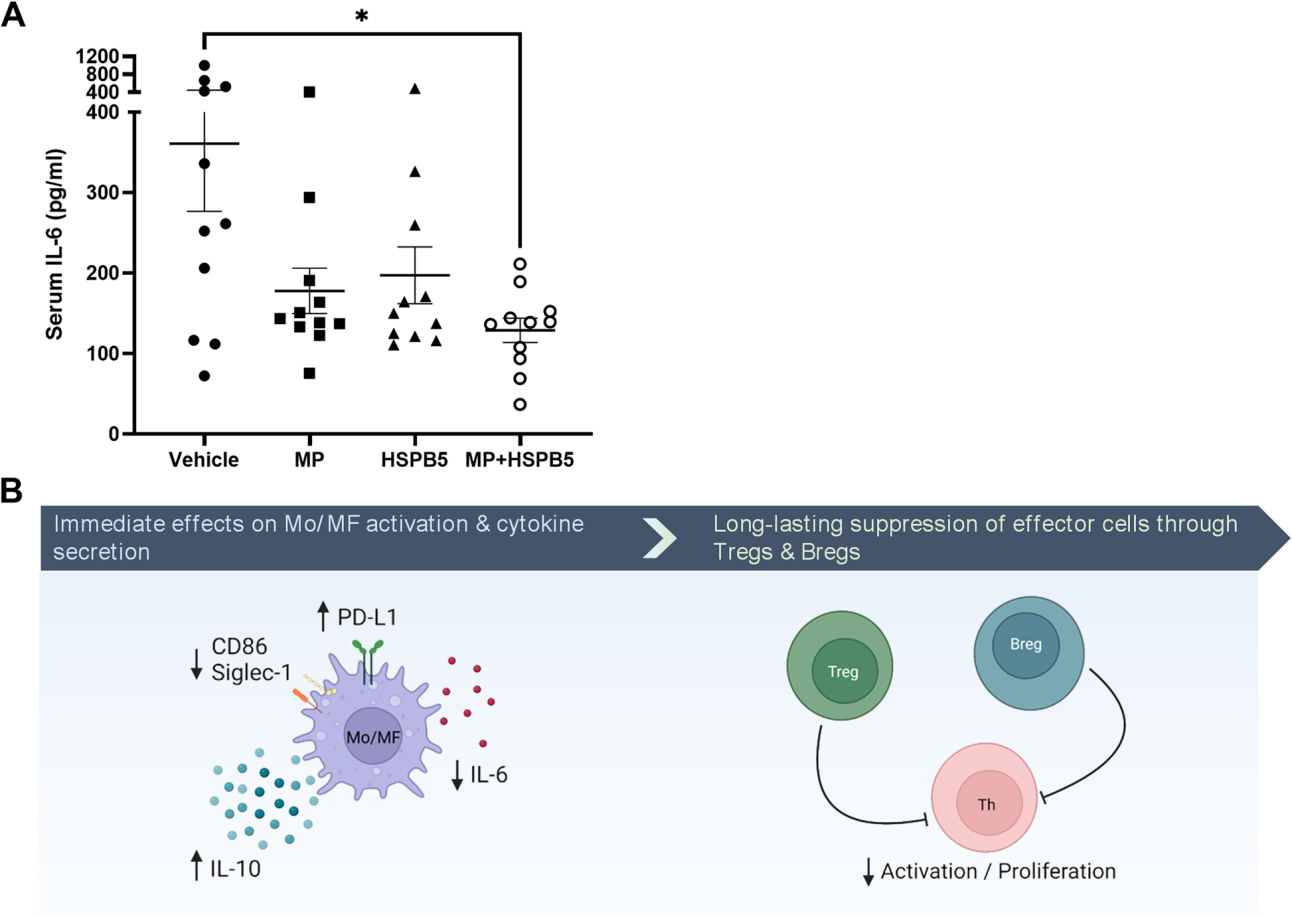
HSPB5 enhances an anti-inflammatory activity of methylprednisolone in an endotoxemia mouse model. Male BALB/c mice at 11 weeks of age were evenly distributed by body weight into 4 groups with 11 mice per group. Endotoxemia was induced by an IP injection of 6 mg/kg LPS (O111:B4). Mice were treated with IP injections of HSPB5 (0.3 mg/kg), methylprednisolone (MP; 1.5 mg/kg), HSPB5 (0.3 mg/kg) + methylprednisolone (1.5 mg/kg), or PBS as a vehicle control 30 min before and 6 h after endotoxemia induction. **A** At 24 h post-endotoxemia induction, terminal blood was collected for IL-6 analysis by ELISA. *p < 0.05. **B** Working model of HSPB5 therapeutic effects. HSPB5 targets inflammatory monocytes/macrophages by binding to TLRs and scavenger receptors. This activates the anti-inflammatory pathways that promote anti-inflammatory cytokine secretion (IL-10; [25]) and suppress pro-inflammatory cytokine secretion (IL-6). Additionally, there is an upregulation of PD-L1 [25]) and downregulation of CD86 and Siglec-1 expression, which contributes to decreased lymphocyte activation or proliferation and increased induction of Tregs and Bregs. Collectively, we propose a model in which HSPB5 immediately alleviates cytokine-mediated inflammation in the kidneys and provides a long-lasting effect on autoimmunity through reduced activation/proliferation of pathogenic Th cells

The side effects from chronic glucocorticoid use contribute significantly to morbidity in LN [31]. In this regard, the steroid-sparing potential of new treatments is an important consideration for LN patient care and drug development. Given the efficacy of HSPB5 in NZB/W F1 mice, we assessed whether coformulation of HSPB5 with a glucocorticoid would enhance the anti-inflammatory effects and therefore provide a rationale for its reduced use when combined with HSPB5. Our initial experiments examined the effect of a matrix of dexamethasone and HSPB5 concentrations on PAM3CSK4-activated THP-1 (a monocytic cell line) cells and showed an enhanced reduction of IL-1β secretion even when HSPB5 was present at low concentrations (Additional file 1: Figure S5). To assess whether HSPB5 could increase the anti-inflammatory effect of a glucocorticoid in vivo, we utilized an LPS-induced endotoxemia mouse model. Our results show that methylprednisolone or HSPB5 each individually reduced the serum level of IL-6 by approximately 50% compared to the vehicle group, although neither treatment was sufficient to achieve a statistically significant reduction (Fig. 6A). However, when mice were treated with co-formulated HSBP5 and methylprednisolone, serum IL-6 levels were significantly reduced by 64% compared to vehicle.

## Discussion

A therapeutic approach that combines immunomodulation with immunosuppression represents an attractive strategy for the treatment of LN. This approach becomes even more attractive if it can reduce the use of glucocorticoids, as their side effects contribute to morbidity. In this report, we demonstrate that HSPB5 can induce regulatory lymphocytes in the spleen, reduce activation of splenic monocytes/macrophages, reduce systemic IL-6, reduce renal infiltration by inflammatory macrophages/monocytes, and ameliorate renal pathology, thus improving the renal function of NZB/W F1 mice. Importantly, the effects of HSPB5 were comparable and, in some instances, superior to methylprednisolone. The results from the LPS-induced endotoxemia study demonstrate that the co-formulation of HSPB5 with methylprednisolone can provide enhanced anti-inflammatory effects, even in the face of a potent and acute inflammatory insult. Taken together, these results suggest that HSPB5 treatment could reduce disease activity, prevent kidney injury, and be steroid-sparing in patients with LN.

HSPB5-treated mice showed substantial improvements in renal pathology and outperformed methylprednisolone in reducing total glomerular and kidney lesions. These results are consistent with our previous study demonstrating improved kidney pathology in MRL/lpr mice treated with HSBP5 [25]. Furthermore, the kidneys of HSPB5-treated mice demonstrated significant preservation of nephrin expression, which suggests improved podocyte integrity and function [32]. Nephrin is a major component of a specialized cell–cell junction formed by podocytes called the slit diaphragm, whose loss is correlated with proteinuria and advanced stages of LN in patients [33]. HSPB5 treatment also significantly reduced the expression of KIM-1, a marker of tubular injury. Urinary KIM-1 is elevated in active LN patients compared to patients in remission and is correlated with pathological tubular atrophy in these patients [34]. Importantly, preservation of the kidney structure with HSPB5 treatment amounted to improved kidney function marked by significantly reduced proteinuria and BUN.

Immune cell infiltration is a hallmark of LN and the nephritic kidney of NZB/W F1 mice. Monocytes are the most abundant infiltrating immune cell in LN patients and the most elevated in NZB/W F1 mice [35, 36]. HSPB5 treatment significantly reduced the renal infiltration of inflammatory monocytes/macrophages (CD11b + Ly6C + F4/80 −), which aligns with the reduced renal expression of key inflammatory mediators of LN progression. For instance, gene expression analyses on sorted immune cells from nephritic kidneys of NZB/W F1 mice showed that BAFF and TNF are predominantly produced by macrophages and dendritic cells and are upregulated during LN onset and progression [37]. IFNγ is an inflammatory cytokine mainly produced by natural killer and T lymphocytes and which acts as a potent activator of macrophages. It has been demonstrated to be upregulated in SLE patient T cells [38] and to contribute to glomerulonephritis in SLE patients [39] as well as the NZB/W F1 [40] and MRL/lpr [41] mouse models. CCL2, one of the key chemokines produced by renal and immune cells in response to kidney injury, was also found to be associated with glomerulonephritis in humans and mice [42]. Taken together, these results demonstrate that HSPB5 treatment was able to preserve renal architecture and function in NZB/W F1 mice by reducing the infiltration of leukocytes and expression of inflammatory cytokines within the kidneys.

Kidney inflammation in LN is initiated by the deposition of immune complexes composed of autoantibodies; macrophages play an essential role in propagating this inflammation and causing renal damage [23, 43]. Peritoneal and splenic macrophages are the main sources of IL-6, which promotes the terminal differentiation of B cells into antibody-secreting plasma cells [29, 44], differentiation of naive CD4 + T cells into Th17 effector cells [45], and suppression of Treg functions [46]. HSPB5 reduced serum IL-6 levels in both the NZB/W F1 and endotoxemia mouse studies, as well as kidney IL-6 transcript levels in NZB/W F1 mice, possibly by modulating both splenic and peritoneal macrophages given the route of administration. We have previously shown that HSPB5 treatment increased IL-10 and PD-L1 expression by splenic macrophages in MRL/lpr mice [25], and here, we demonstrated that HSPB5 significantly lowered the expression of CD86 on splenic monocytes/macrophages, suggesting reduced activation of these cells. Our study is consistent with those from others showing that lower IL-6 contributes to improvements in proteinuria and survival in lupus mouse models [29]. Although we observed a very slight reduction in the generation of anti-dsDNA antibodies early in the study, the effect was not maintained. Since HSPB5 dampens monocyte/macrophage-dependent inflammatory responses, combining HSPB5 treatment with an anti-CD20 antibody such as rituximab or obinutuzumab, which have been shown to reduce anti-dsDNA antibody titers and cause B cell depletion in LN patients [47, 48], represents an attractive strategy to improve renal response rates.

HSPB5 treatment also had a profound effect on the induction of Tregs and Bregs in the spleen of NZB/W F1 mice. Tregs limit T effector cell proliferation by consuming local IL-2, secreting suppressive mediators such as IDO, IL-10, and TGFβ; by direct killing through granzyme B secretion; and by inhibiting the stimulatory capacity of APCs [49–51]. Bregs, such as B-1a (CD5 + CD19 +) cells, negatively regulate Th cell responses and promote induction of Tregs through secretion of IL-10 [52–54]. Here, we demonstrated that HSPB5 significantly increased Treg and Breg frequency in the spleens of NZB/W mice. Additionally, our previous work showed that HSPB5 treatment slightly increased T and B regulatory cell frequency in the spleens of MRL/lpr mice [25]. The frequency of Tregs was also modestly elevated in the kidneys of these mice after treatment with HSPB5, possibly due to the increased induction of these cells in the spleen. Thus, we demonstrated via two independent studies and two different preclinical efficacy models that HSPB5 intercepts splenic monocyte/macrophage-dependent inflammatory responses and T effector cells before they are mobilized into the kidneys of lupus-prone mice. Others have shown that adoptive transfers of Tregs delay progression of proteinuria and improve renal histology in NZB/W F1 mice [55, 56]. Similarly, the adoptive transfer of Bregs (CD1d + CD5 + B cells) into CD19 − / − NZB/W F1 mice restored splenic Tregs and delayed the development of nephritis [57]. The induction of regulatory lymphocytes may also help explain the long-lasting effects of HSPB5 in our studies and in human subjects, despite its short half-life [58].

When LN becomes clinically evident due to intrarenal inflammation and cellular injury, the autoimmune processes leading to these events have already been active for some time [59, 60]. High-dose methylprednisolone is given for the first few days during a renal flare to lower inflammation and preserve kidney function. However, disease remains active on a cellular and molecular level longer than is clinically observable [59, 60]. Cytokines, complement, immune complexes, and inflammatory leukocytes continue the inflammatory process, leading to future LN clinical activity. Thus, treatments targeting autoimmunity should follow the suppression of inflammation to prevent the reactivation of LN [4]. Voclosporin and belimumab targeting T and B lymphocytes, respectively, are the only approved anti-autoimmunity therapies for LN. As illustrated in our working model (Fig. 6B), we believe that HSPB5 is an excellent candidate for clinical development given its dual mechanism of action targeting intrarenal inflammation through the reduction of renal immune cell infiltration, and lymphocyte activity through the induction of regulatory T and B cells. We have demonstrated in two well-established models of LN that HSPB5 is able to reduce kidney damage when administered after disease onset, closely mimicking possible future conditions on how this therapy could benefit LN patients. Having been demonstrated safe and well-tolerated in phase I and IIa clinical trials (phase I: NCT02442557; phase IIa: NCT02442570), HSPB5 is in good standing to potentially address the severe pathology of LN while also reducing the use of glucocorticoids.

## Supplementary information

**Additional file 1.**

## Data Availability

The datasets used and/or analyzed during the current study are available from the corresponding author upon reasonable request.

## Abbreviations

AHT: Animal health technician
APC: Antigen-presenting cells
BUN: Blood urea nitrogen
H&E: Hematoxylin and eosin
HSPB5: ɑB-crystallin
LN: Lupus nephritis
LPS: Lipopolysaccharide
MMF: Mycophenolate mofetil
PAS: Periodic acid–Schiff
SEM: Standard error of the mean
SLE: Systemic lupus erythematosus
TLR: Toll-like receptor

## References

[1] Brunner HI, Gladman DD, Ibañez D, Urowitz MD, Silverman ED (2008). Difference in disease features between childhood-onset and adult-onset systemic lupus erythematosus. Arthritis Rheum.

[2] Dörner T, Furie R (2019). Novel paradigms in systemic lupus erythematosus. The Lancet.

[3] Tektonidou MG, Dasgupta A, Ward MM (2016). Risk of end-stage renal disease in patients with lupus nephritis, 1971–2015: a systematic review and Bayesian meta-analysis. Arthritis Rheumatol Hoboken NJ.

[4] Rovin BH, Parikh SV (2014). Lupus nephritis: the evolving role of novel therapeutics. Am J Kidney Dis Off J Natl Kidney Found.

[5] Horwitz J (1992). Alpha-crystallin can function as a molecular chaperone. Proc Natl Acad Sci U S A.

[6] Cetinel S, Semenchenko V, Cho JY, Sharaf MG, Damji KF, Unsworth LD (2017). UV-B induced fibrillization of crystallin protein mixtures. PLoS ONE.

[7] Srinivas V, Raman B, Rao KS, Ramakrishna T, Rao CM (2003). Structural perturbation and enhancement of the chaperone-like activity of alpha-crystallin by arginine hydrochloride. Protein Sci Publ Protein Soc.

[8] Sreekumar PG, Kannan R, Kitamura M, Spee C, Barron E, Ryan SJ (2010). αB crystallin is apically secreted within exosomes by polarized human retinal pigment epithelium and provides neuroprotection to adjacent cells. Blagosklonny MV, editor. PLoS ONE.

[9] Gangalum RK, Atanasov IC, Zhou ZH, Bhat SP (2011). αB-crystallin is found in detergent-resistant membrane microdomains and is secreted via exosomes from human retinal pigment epithelial cells *. J Biol Chem.

[10] Hunter-Lavin C, Davies EL, Bacelar MMFVG, Marshall MJ, Andrew SM, Williams JHH (2004). Hsp70 release from peripheral blood mononuclear cells. Biochem Biophys Res Commun.

[11] Bsibsi M, Holtman IR, Gerritsen WH, Eggen BJL, Boddeke E, van der Valk P (2013). Alpha-B-crystallin induces an immune-regulatory and antiviral microglial response in preactive multiple sclerosis lesions. J Neuropathol Exp Neurol.

[12] van Noort JM, Bsibsi M, Nacken PJ, Gerritsen WH, Amor S, Holtman IR (2013). Activation of an immune-regulatory macrophage response and inhibition of lung inflammation in a mouse model of COPD using heat-shock protein alpha B-crystallin-loaded PLGA microparticles. Biomaterials.

[13] Jin C, Cleveland JC, Ao L, Li J, Zeng Q, Fullerton DA (2014). Human myocardium releases heat shock protein 27 (HSP27) after global ischemia: the proinflammatory effect of extracellular HSP27 through Toll-like receptor (TLR)-2 and TLR4. Mol Med.

[14] Vabulas RM, Braedel S, Hilf N, Singh-Jasuja H, Herter S, Ahmad-Nejad P (2002). The endoplasmic reticulum-resident heat shock protein Gp96 activates dendritic cells via the Toll-like receptor 2/4 pathway. J Biol Chem.

[15] Chebotareva N, Bobkova I, Shilov E (2017). Heat shock proteins and kidney disease: perspectives of HSP therapy. Cell Stress Chaperones.

[16] Iwaki T, Kume-Iwaki A, Goldman JE (1990). Cellular distribution of alpha B-crystallin in non-lenticular tissues. J Histochem Cytochem.

[17] Dubin RA, Wawrousek EF, Piatigorsky J (1989). Expression of the murine alpha B-crystallin gene is not restricted to the lens. Mol Cell Biol.

[18] Guo YS, Liang PZ, Lu SZ, Chen R, Yin YQ, Zhou JW (2019). Extracellular αB-crystallin modulates the inflammatory responses. Biochem Biophys Res Commun.

[19] Arac A, Brownell SE, Rothbard JB, Chen C, Ko RM, Pereira MP (2011). Systemic augmentation of αB-crystallin provides therapeutic benefit twelve hours post-stroke onset via immune modulation. Proc Natl Acad Sci.

[20] Klopstein A, Santos-Nogueira E, Francos-Quijorna I, Redensek A, David S, Navarro X (2012). Beneficial effects of αB-crystallin in spinal cord contusion injury. J Neurosci Off J Soc Neurosci.

[21] Ousman SS, Tomooka BH, van Noort JM, Wawrousek EF, O’Conner K, Hafler DA (2007). Protective and therapeutic role for αB-crystallin in autoimmune demyelination. Nature.

[22] Rothbard JB, Kurnellas MP, Brownell S, Adams CM, Su L, Axtell RC (2012). Therapeutic effects of systemic administration of chaperone αB-crystallin associated with binding proinflammatory plasma proteins*. J Biol Chem.

[23] Ma C, Xia Y, Yang Q, Zhao Y (2019). The contribution of macrophages to systemic lupus erythematosus. Clin Immunol.

[24] Labonte AC, Kegerreis B, Geraci NS, Bachali P, Madamanchi S, Robl R (2018). Identification of alterations in macrophage activation associated with disease activity in systemic lupus erythematosus. PLoS ONE.

[25] Berg SIT, Knapp J, Braunstein M, Shirriff C (2022). The small heat shock protein HSPB5 attenuates the severity of lupus nephritis in lupus-prone mice. Autoimmunity.

[26] Alperovich G, Rama I, Lloberas N, Franquesa M, Poveda R, Gomà M (2007). New immunosuppresor strategies in the treatment of murine lupus nephritis. Lupus.

[27] Livak KJ, Schmittgen TD (2001). Analysis of relative gene expression data using real-time quantitative PCR and the 2^−ΔΔCT^ method. Methods San Diego Calif.

[28] Haselmayer P, Vigolo M, Nys J, Schneider P, Hess H (2017). A mouse model of systemic lupus erythematosus responds better to soluble TACI than to soluble BAFFR, correlating with depletion of plasma cells. Eur J Immunol.

[29] Tackey E, Lipsky P, Illei G (2004). Rationale for interleukin-6 blockade in systemic lupus erythematosus. Lupus.

[30] Kawachi H, Fukusumi Y (2020). New insight into podocyte slit diaphragm, a therapeutic target of proteinuria. Clin Exp Nephrol.

[31] Mejía-Vilet JM, Ayoub I (2021). The use of glucocorticoids in lupus nephritis: new pathways for an old drug. Front Med.

[32] Perysinaki GS, Moysiadis DK, Bertsias G, Giannopoulou I, Kyriacou K, Nakopoulou L (2011). Podocyte main slit diaphragm proteins, nephrin and podocin, are affected at early stages of lupus nephritis and correlate with disease histology. Lupus.

[33] Tian Y, Guo H, Miao X, Xu J, Yang R, Zhao L (2020). Nestin protects podocyte from injury in lupus nephritis by mitophagy and oxidative stress. Cell Death Dis.

[34] Ding Y, Nie LM, Pang Y, Wu WJ, Tan Y, Yu F (2018). Composite urinary biomarkers to predict pathological tubulointerstitial lesions in lupus nephritis. Lupus.

[35] Cao Y, Tang W, Tang W (2019). Immune cell infiltration characteristics and related core genes in lupus nephritis: results from bioinformatic analysis. BMC Immunol.

[36] Li B, Tang Y, Ni X, Chen W (2020). Immune cell landscape identification associates intrarenal mononuclear phagocytes with onset and remission of lupus nephritis in NZB/W mice. Front Genet.

[37] Schiffer L, Bethunaickan R, Ramanujam M, Huang W, Schiffer M, Tao H (2008). Activated renal macrophages are markers of disease onset and disease remission in lupus nephritis. J Immunol Baltim Md 1950.

[38] Harigai M, Kawamoto M, Hara M, Kubota T, Kamatani N, Miyasaka N (2008). Excessive production of IFN-gamma in patients with systemic lupus erythematosus and its contribution to induction of B lymphocyte stimulator/B cell-activating factor/TNF ligand superfamily-13B. J Immunol Baltim Md 1950.

[39] Uhm W-S, Na K, Song G-W, Jung S-S, Lee T, Park M-H (2003). Cytokine balance in kidney tissue from lupus nephritis patients. Rheumatology.

[40] Haas C, Ryffel B, Le Hir M (1998). IFN-gamma receptor deletion prevents autoantibody production and glomerulonephritis in lupus-prone (NZB x NZW)F1 mice. J Immunol Baltim Md 1950.

[41] Schwarting A, Wada T, Kinoshita K, Tesch G, Kelley VR (1998). IFN-gamma receptor signaling is essential for the initiation, acceleration, and destruction of autoimmune kidney disease in MRL-Fas(lpr) mice. J Immunol Baltim Md 1950.

[42] Vielhauer V, Anders HJ, Schlöndorff D (2007). Chemokines and chemokine receptors as therapeutic targets in lupus nephritis. Semin Nephrol.

[43] Davidson A (2016). What is damaging the kidney in lupus nephritis?. Nat Rev Rheumatol.

[44] Alarcón-Riquelme ME, Möller G, Fernández C (1993). Macrophage depletion decreases IgG anti-DNA in cultures from (NZB x NZW)F1 spleen cells by eliminating the main source of IL-6. Clin Exp Immunol.

[45] Kimura A, Kishimoto T (2010). IL-6: regulator of Treg/Th17 balance. Eur J Immunol.

[46] Parietti V, Monneaux F, Décossas M, Muller S (2008). Function of CD4+, CD25+ Treg cells in MRL/lpr mice is compromised by intrinsic defects in antigen-presenting cells and effector T cells. Arthritis Rheum.

[47] Rovin BH, Furie R, Latinis K, Looney RJ, Fervenza FC, Sanchez-Guerrero J (2012). Efficacy and safety of rituximab in patients with active proliferative lupus nephritis: the Lupus Nephritis Assessment with Rituximab study. Arthritis Rheum.

[48] Furie RA, Aroca G, Cascino MD, Garg JP, Rovin BH, Alvarez A (2022). B-cell depletion with obinutuzumab for the treatment of proliferative lupus nephritis: a randomised, double-blind, placebo-controlled trial. Ann Rheum Dis.

[49] Liu Z, Gerner MY, Van Panhuys N, Levine AG, Rudensky AY, Germain RN (2015). Immune homeostasis enforced by co-localized effector and regulatory T cells. Nature.

[50] Schmidt A, Oberle N, Krammer PH (2012). Molecular mechanisms of treg-mediated T cell suppression. Front Immunol.

[51] Mizui M, Tsokos GC (2018). Targeting regulatory T cells to treat patients with systemic lupus erythematosus. Front Immunol.

[52] O’Garra A, Chang R, Go N, Hastings R, Haughton G, Howard M (1992). Ly-1 B (B-1) cells are the main source of B cell-derived interleukin 10. Eur J Immunol.

[53] Mauri C, Menon M (2015). The expanding family of regulatory B cells. Int Immunol.

[54] Matsushita T (2019). Regulatory and effector B cells: friends or foes?. J Dermatol Sci.

[55] Scalapino KJ, Tang Q, Bluestone JA, Bonyhadi ML, Daikh DI (2006). Suppression of disease in New Zealand Black/New Zealand white lupus-prone mice by adoptive transfer of ex vivo expanded regulatory T cells. J Immunol.

[56] Weigert O, von Spee C, Undeutsch R, Kloke L, Humrich JY, Riemekasten G (2013). CD4+Foxp3+ regulatory T cells prolong drug-induced disease remission in (NZBxNZW) F1 lupus mice. Arthritis Res Ther.

[57] Watanabe R, Ishiura N, Nakashima H, Kuwano Y, Okochi H, Tamaki K (2010). Regulatory B cells (B10 cells) have a suppressive role in murine lupus: CD19 and B10 cell deficiency exacerbates systemic autoimmunity. J Immunol Baltim Md 1950.

[58] van Noort JM, Bsibsi M, Nacken PJ, Verbeek R, Venneker EHG (2015). Therapeutic intervention in multiple sclerosis with alpha B-crystallin: a randomized controlled phase IIa trial. Wiendl H, editor. PLOS ONE..

[59] Tsokos GC, Lo MS, Reis PC, Sullivan KE (2016). New insights into the immunopathogenesis of systemic lupus erythematosus. Nat Rev Rheumatol.

[60] Yu F, Haas M, Glassock R, Zhao MH (2017). Redefining lupus nephritis: clinical implications of pathophysiologic subtypes. Nat Rev Nephrol.

