# Supplementary material for: HSPB5 suppresses renal inflammation and protects lupus-prone NZB/W F1 mice from severe renal damage
Source: Arthritis Res Ther. 2022 Dec 12;24:267. doi: 10.1186/s13075-022-02958-9 (PMC9743758; doi:10.1186/s13075-022-02958-9)
Supplement: Supplementary file 1 — Additional file 1. [file 13075_2022_2958_MOESM1_ESM.pdf]

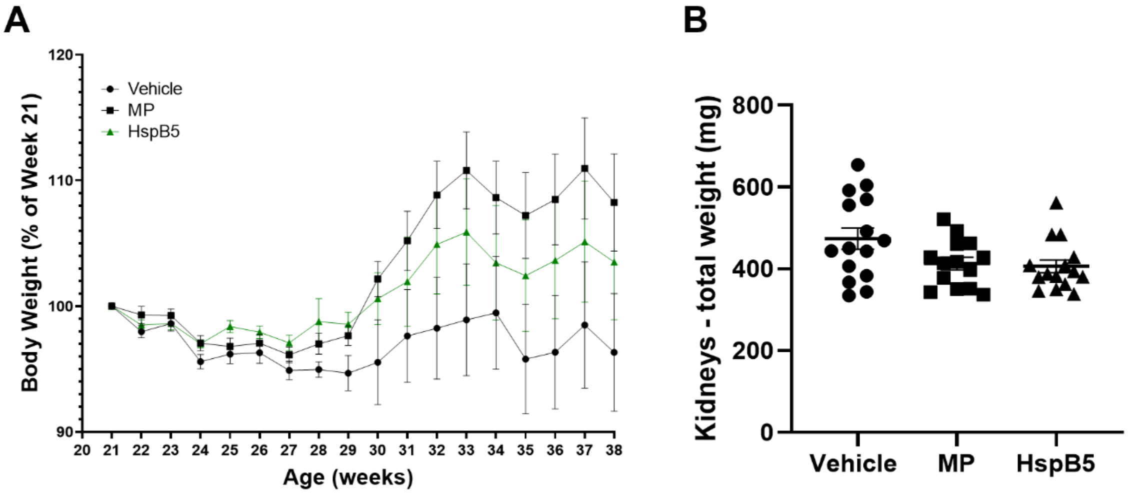

Supplementary Figure 1. 23-week-old female NZB/W F1 mice were treated three times per week intraperitoneally with 0.3 mg/kg HSPB5, 3 mg/kg methylprednisolone (MP), or vehicle until week 38 (n=14-15 per group). A) Body weight was recorded weekly. B) At 38 weeks, mice were sacrificed for tissue collection. The weight of the left and right kidney were combined and recorded as total kidney weight. Data are presented as means ± SEM.

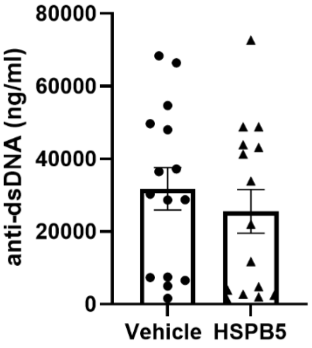

Supplementary Figure 2. 23-week-old female NZB/W F1 mice were treated three times per week intraperitoneally with 0.3 mg/kg HSPB5 or vehicle (n=14-15 per group). At 27 weeks of age a blood sample was collected and serum anti-dsDNA antibody levels were determined by ELISA. Data are presented as means ± SEM.

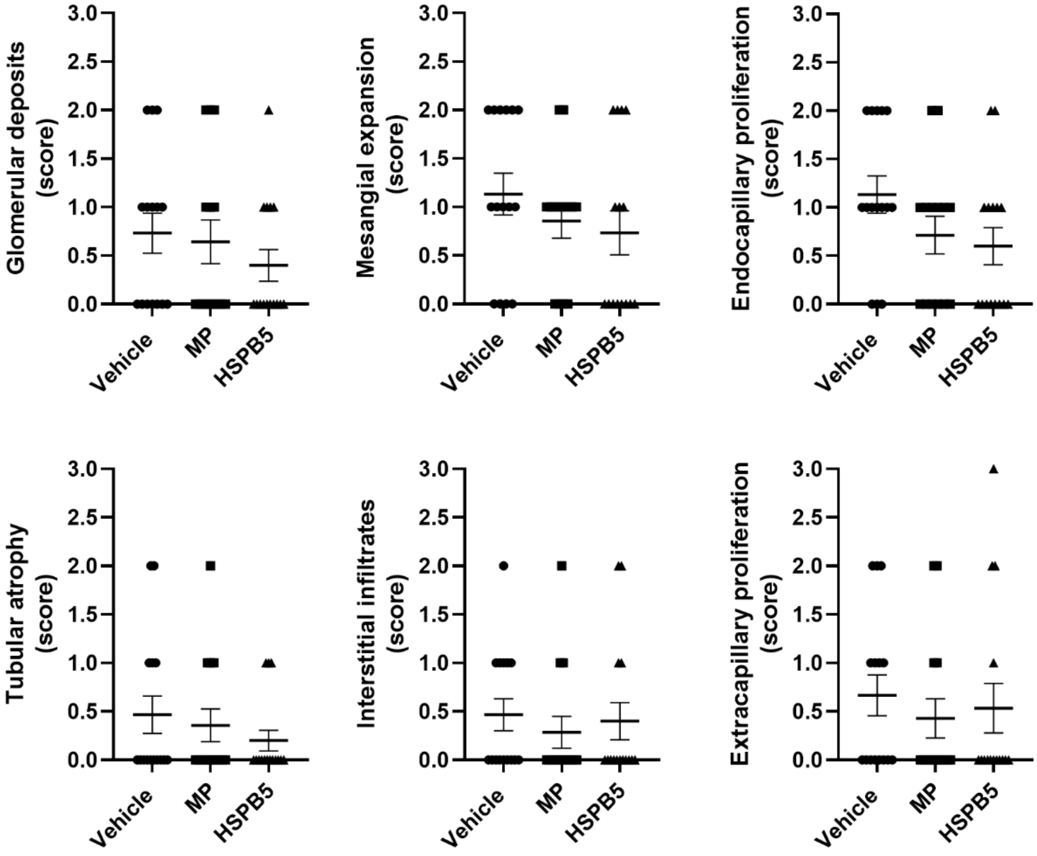

Supplementary Figure 3. 23-week-old female NZB/W F1 mice were treated three times per week intraperitoneally with 0.3 mg/kg HSPB5, 3 mg/kg methylprednisolone (MP), or vehicle until week 38 (n=14-15 per group). At 38 weeks, mice were sacrificed for tissue collection. Kidneys were formalin-fixed, paraffin-embedded (FFPE) and stained with periodic acid–Schiff (PAS) prior to evaluation by a pathologist blinded to the study. Data are presented as means ± SEM.

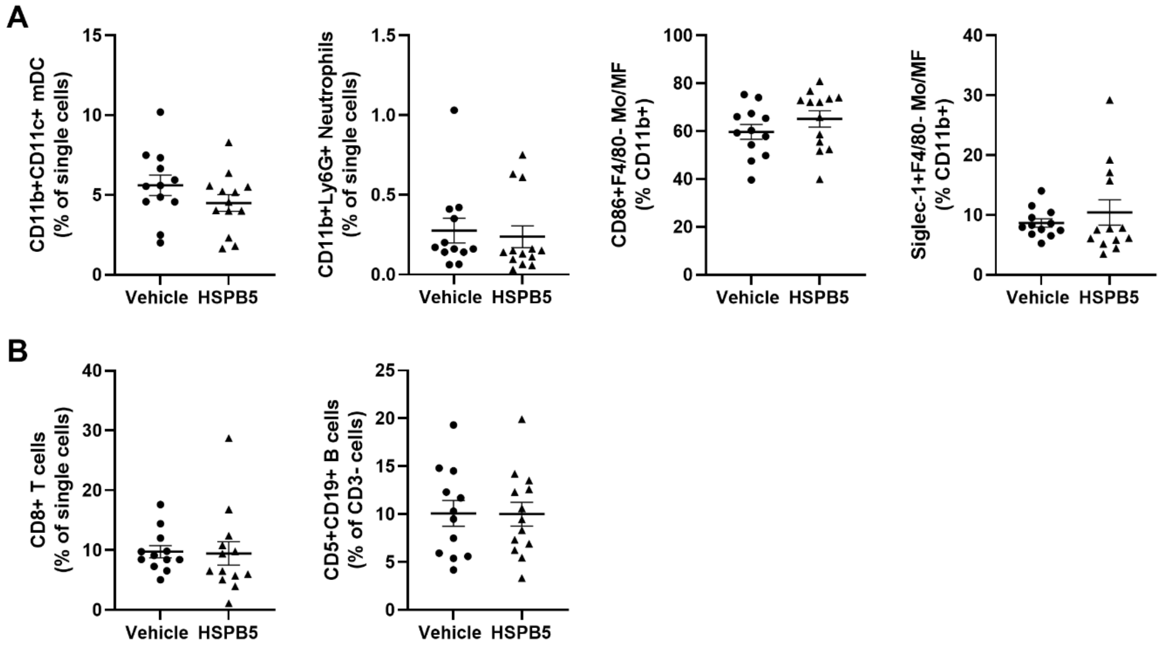

Supplementary Figure 4. 23-week-old female NZB/W F1 mice were treated three times per week intraperitoneally with 0.3 mg/kg HSPB5, 3 mg/kg methylprednisolone (MP), or vehicle until week 38 (n=14-15 per group). At 38 weeks, mice were sacrificed for tissue collection. Kidneys were processed to single cell suspensions, stained, and analyzed by flow cytometry. A) Flow cytometric analysis of myeloid populations defined as mDC (CD11b+CD11c+), neutrophils (CD11b+Ly6G+) and activated macrophages (CD86+ or Siglec-1+ shown as percentage of CD11b+ cells). B) Flow cytometric analysis of lymphocyte populations defined as total cytotoxic T cells (CD8+) and Bregs (CD5+CD19+ shown as percent of lineage negative (CD3-) cells). Data are presented as means ± SEM.

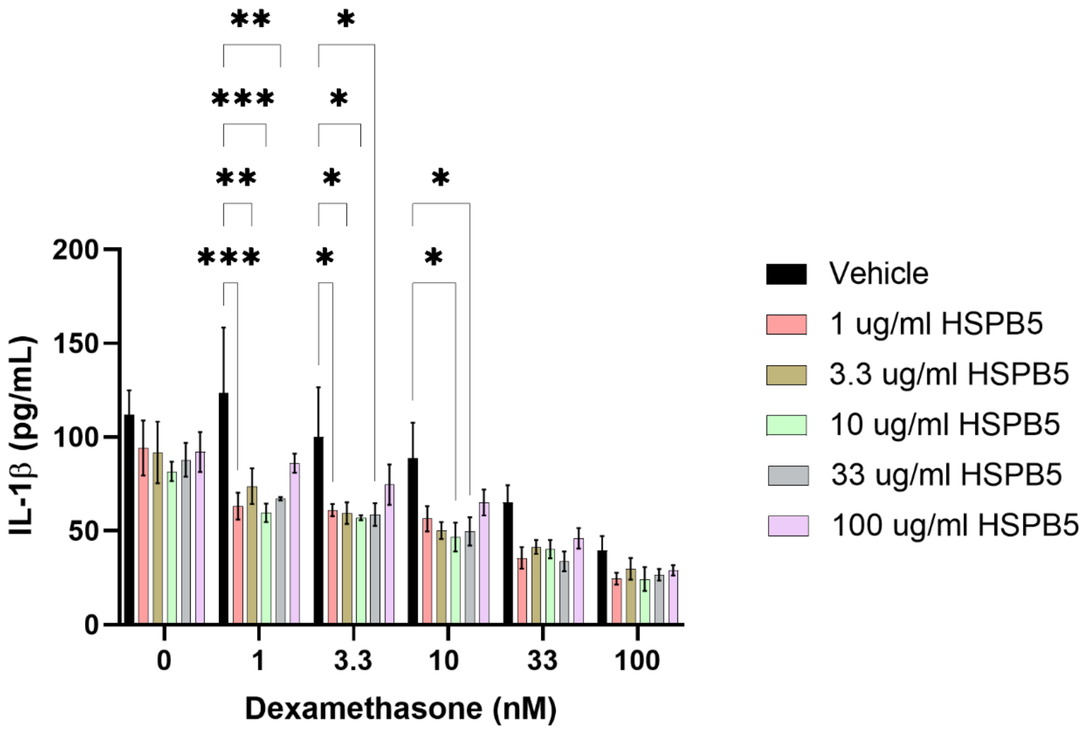

Supplementary Figure 5. THP-1 cells were seeded at 1 x 10^6 cells/mL and pre-treated with dexamethasone (DEX), HSPB5, or HSPB5 + DEX for 1h. Cells were then washed and activated with 100 ng/mL Pam3CSK4 (R&D Systems) for 20h. Cells were treated for 1h with 5mM ATP (Sigma-Aldrich) before collecting conditioned media for cytokine analysis. Conditioned media was stored at -20°C until analysis. Results shown are the average of 3 independent experiments. Data are presented as means ± SEM. *indicates $p<0.05$. ** $p<0.01$. ***$p<0.001$.
